# Seasonality of Tropical Instability Waves and Its Feedback to the Seasonal Cycle in the Tropical Eastern Pacific

**DOI:** 10.1100/2012/612048

**Published:** 2012-11-11

**Authors:** Seul-Hee Im, Soon-Il An, Matthieu Lengaigne, Yign Noh

**Affiliations:** ^1^Department of Atmospheric Sciences, Yonsei University, Seoul 120-749, Republic of Korea; ^2^Institut de Recherche pour le Developpement, Laboratoire d'Oceanographie et de Climatologie, Experimentations et Approches Numériques, Couloir 45-55, 4ème étage, Case 100, 4 place Jussieu, 75252 Paris Cedex 05, France

## Abstract

This study investigated the seasonality of tropical instability waves (TIWs) and its feedback to the seasonal cycle in the tropical eastern Pacific using a high-resolution ocean model covering 1958–2007. The climatological mean of the TIWs featured intraseasonal fluctuations, implying that TIWs are not occurring randomly, but their amplitude is partly in phase from one year to another. This seasonality of TIW activity is attributed to their dependency on the seasonal mean variation of current and temperature. Energy conversion analysis confirmed that the strong variability of TIWs near 4°N was due to the barotropic energy conversion associated with the large meridional shear of NECC and SEC and that at another pole near 2°N was due to the baroclinic energy conversion associated with the temperature front in the mixed layer. The former and latter poles are somehow largely responsible for amplifying the dynamic and thermal eddies of TIWs, respectively. The intensified TIWs during a boreal fall increase the tropical eastern Pacific SST by associating the warm thermal advection by anomalous currents, with a rate of up to 1°C/month in September. Therefore, this leads to interactive feedback between seasonal and intraseasonal variations, that is, TIWs in the tropical eastern Pacific.

## 1. Introduction

Tropical instability waves (TIWs) are intraseasonal fluctuations observed both in the Atlantic and Pacific oceans. They are easily observed in satellite images of sea surface temperature (SST) and ocean color. In the Pacific Ocean, for example, they are clearly seen between 160° and 90°W and 4°S-4°N as cusp-like features [1]. TIWs are westward-propagating waves with a wavelength of 1000–2000 km and a 20–40-day period [2, 3].

Early numerical studies [4, 5] demonstrated that TIWs arise from barotropic instability due to shears between the equatorial undercurrent (EUC) and south equatorial current (SEC), as well as between the SEC and north equatorial countercurrent (NECC). Other idealized numerical-modeling studies argued that baroclinic [6] and frontal [7] instabilities also contribute to the generation of TIWs. An observation study by Grodsky et al., 2005 [8] showed that the intensity of TIWs in the equatorial Atlantic is in phase with the strengthening of trade winds and the cold tongue. They also found that TIWs are maintained by barotropic and baroclinic energy conversion using mooring data at 0°N, 23°W. 

Since both barotropic and baroclinic instabilities are determined by climate conditions, the activity of TIWs is presumably related to both annual and interannual variations in the tropical climate state. For example, Von Schuckmann et al., 2008 [9], using an ocean model, showed that the rate of kinetic energy production by TIWs follows a seasonal change in the shear of the equatorial zonal current. They also suggested that the seasonal modulation of the TIWs is more dominated by the seasonality of the NECC than EUC or SEC in the tropical Atlantic. As another example, the seasonal or interannual variations in the cold tongue influence TIW activity such that the intensified cold tongue creates potentially strong baroclinic instability, leading to active TIWs [10].

As mentioned above, TIW activity depends on the climate state. However, a TIW in turn may influence the climate state by modifying eddy heat flux. For example, TIWs are most active during boreal fall [10] when the cold tongue is fully developed. A TIW produces the strongest warming effect during this season, implying a negative feedback to cold tongue intensity. Thus, it is expected that TIWs could modulate equatorial SST. Some analyses of the upper-ocean heat balance using observed data actually showed that the horizontal heat flux induced by TIWs is sometimes equivalent to the effects of seasonal forcing itself [11–13]. Using observational data, Jochum et al., 2007 [14] also computed the horizontal temperature advection by TIWs, which was 2.8°C/month at 0°N, 110°W and 0.8°C/month at 0°N, 140°W during the years 2002 to 2005. According to their estimation, 25% of heating is attributed to zonal temperature advection. Menkes et al., 2006 [15] also estimated the horizontal temperature advection by TIWs, which was 0.84°C/month in the equatorial eastern Pacific (2°S–6°N, 160°–90°W) and showed that vertical temperature advection is negligible in driving SST anomalies. So far, most studies of the effect of TIWs on the SST field have not dealt with data over a longer time period, and, furthermore, they are based only on horizontal advection and rarely include vertical advection due to TIWs because of data limitations. However, below the mixed layer, vertical temperature advection by TIWs is supposed to cause significant cooling. 

To provide better insight into the seasonality of TIWs and their impact on the seasonal cycle, we investigated the seasonal amplitude-locking of TIWs in three-dimensional space and their feedback to the climatological climate states. In particular, the long-term averaged climatological features of a TIW will reveal how strictly the activities of TIWs are modulated by the seasonal cycle. In Section 2, the data utilized in this study are introduced.  Section 3 discusses the seasonality of a TIW obtained from the long-term averaged climatological mean and addresses to what extent a TIW is seasonally amplitude-locked and what its main features are. The relative features between the barotropic and baroclinic energy conversions of a TIW are also provided in this same section. The feedback of TIWs to the mean state through eddy heat flux is presented in Section 4. Summary and concluding remarks are given in Section 5. 

## 2. Data

Because of their small spatial and temporal scales, studies involving TIWs require high-resolution data. Because fine-resolution three-dimensional observational data are scarce, modeling offers a promising alternative to perform such an investigation. In this study, we used five-day mean ocean currents, temperature, and density obtained from the ocean general circulation model.

The numerical simulations analyzed in this study are part of the Drakkar hierarchy of global configurations [16] and are detailed in the simulation by [17]. The model configuration used in this study was built from the “Nucleus for European Modeling of the Ocean” ocean/sea-ice numerical framework (NEMO v3.2, [18]), comprising the ocean model formerly known as OPA, coupled to the Louvain-la-Neuve sea-ice model (LIM) [19]. This ocean general circulation model (OGCM) has been extensively validated in the uncoupled mode [20–22] and coupled mode [23] in the tropical Pacific where it succeeds in reproducing the basin-wide structures of currents, sea level, and temperature and accurately simulates equatorial dynamics.

The configuration used in this study (known as ORCA025) employs a tripolar, quasi-isotropic grid with a nominal resolution of 1/4° (i.e., cell size ~25 km in the tropics). Forty-six vertical levels were used, with ten levels in the upper 100 m and 250 m resolution for depth. The mixed layer dynamics were parameterized using a turbulent kinetic energy (TKE) closure scheme [24], allowing for high values in the surface boundary layer as well as a minimum value of 10^−5 ^m^2^ s^−1^ in the thermocline. Additional subgrid-scale mixing parameterizations included bi-Laplacian viscosity and iso-neutral Laplacian diffusivity. For tracer advection, a total variance dissipation scheme—a second-order, two-step monotonic scheme with moderate numerical diffusion—was used [25].

In this study, the model was forced from 1958 to 2007 with Drakkar Forcing Set #3 (DFS3), described in detail by [17]. The starting point of DFS3 is the CORE dataset developed by [26] and is used to intercompare various global ocean components of a coupled system [27]. To calculate latent and sensible heat fluxes, the CORE bulk formulae algorithm was used, with surface atmospheric state variables derived from ERA40 reanalysis and ECMWF analysis after 2002 (air temperature, humidity, and winds at 10 m). These selected input fields were corrected for temporal discontinuities to yield better agreement with some recent high quality data. Radiation fluxes were based on the CORE v1 dataset, using a corrected ISCCP-FD radiation product [28] available from 1984. Precipitation was derived from the GXGXS dataset [26], based on the blending of several existing products from 1979. Climatology was imposed before 1979. No surface temperature restoration was performed, and salinity restoration, corresponding to a relaxation time scale of 33 days for 10 m, was used even under sea ice. 

To check how well the model simulated the observed TIW features, model data was compared to the observed SST from the Tropical Rainfall Measuring Mission (TRMM) Microwave Imager (TMI). We utilized TMI optimally interpolated SST (OI SST) version-3 data for the January 1998–December 2010 period (http://www.ssmi.com/). For the analysis of TIWs, a 50-day high-pass-filter was applied to observational and model data. Hereafter, TIWs used in our analysis indicate 50-day high-pass-filtered components. In this study, we focused on the climatological cycle of TIWs over the eastern Pacific. The climatological cycle can be defined as the average of 50-year data at each pentad of calendar days. Thereby, a possible interannual impact on TIWs to a large extent was removed. 

**Figure 1:**
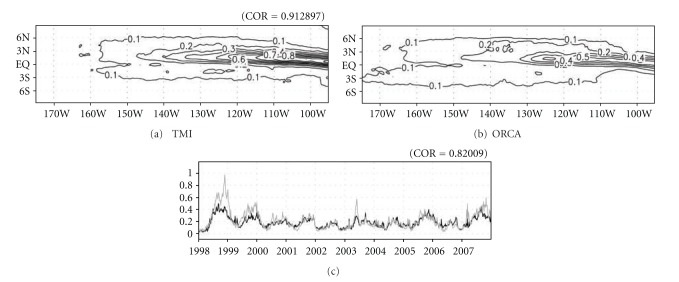
Temporal variance of SST from (a) TMI and (b) ORCA model data for ten years, and (c) time-varying spatial variance over 160–90°W, 4°S-4°N for the TMI (black curve) and ORCA model (gray curve).

The activity of TIWs in the model was compared with observations. The activity of TIWs was defined by a temporal variance of SST for ten years (January 1998–December 2007) and the time-varying spatial variance over 160°W–90°W, 4°S-4°N. In Figures 1(a) and 1(b), the distributions of temporal variance in the model TIWs closely parallel the ones based on observation, and they had a high correlation of 0.91. The locations of maximum variance between 140°W–90°W near 1°N in the model and observations coincided [1]. The time series of spatial variance in the model TIWs were also similar to that from observations (Figure 1(c)). Their correlation was 0.82. These comparisons reveal that the model simulated the observed TIWs' properties reasonably well. The reproduced amplitude of TIWs, however, was slightly weaker in the model. 

## 3. Seasonality of TIWs

**Figure 2:**
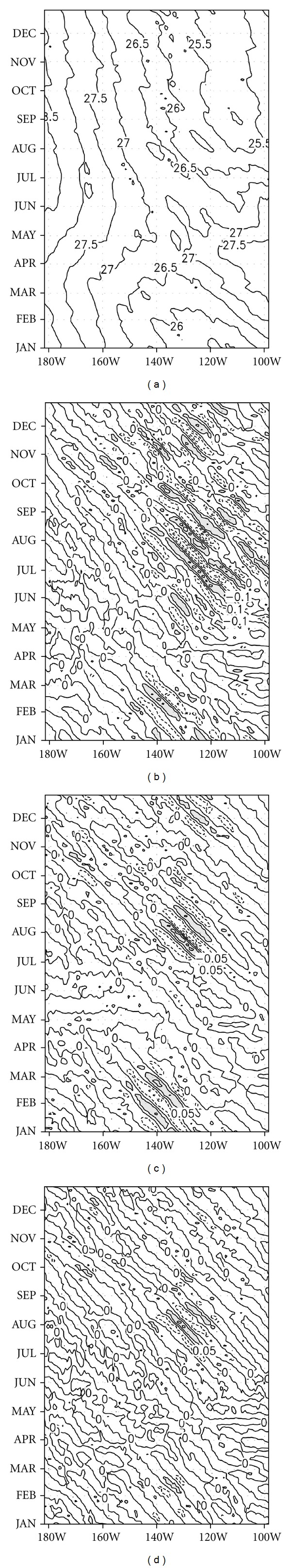
Longitude-time plot of the 50-year climatological-mean (1998–2007) at 2°N for (a) SST [°C] and 50-day high-pass filtered (b) temperature [°C], (c) zonal current [m/s], and (d) meridional current [m/s].

The seasonality of TIWs was examined in the longitudinal climatological cycle for 50 years at 2°N where the amplitudes of TIWs were large.  Figure 2(a) shows the climatological cycle of SST. As seen in Figure 2(a), the seasonal cycle of the cold tongue is distinctly visible with its coldest period appearing in the fall season. Figures 2(b), 2(c), and 2(d) show the climatological cycle of TIWs in temperature, zonal current, and meridional current, respectively, averaged from the surface to a depth of 50 m. The pattern of TIWs was more prominent in temperature than currents. TIWs started to appear in June near 110°W, propagated westward, and continued until the end of the year. The seasonality of TIWs was apparent in the 50-year averaged climatological cycle.

**Figure 3:**
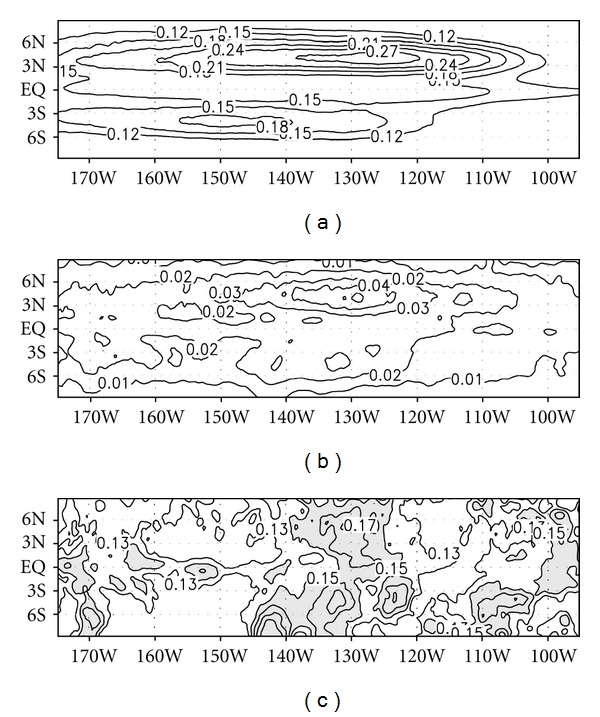
Distributions of the root-mean-square of TIWs computed from (a) 50-year data and (b) climatological-mean for 50 years. The ratio between the two figures, (b)/(a), is represented in (c). TIWs were defined as the 50-day high-pass filtered meridional current averaged from the surface to a depth of 50 m. Units for (a) and (b) are [m/s]^2^.

The level of variability of TIWs based on 50-year data and the associated climatological cycle were investigated. The level of variability was estimated by calculating the root-mean-square of the filtered meridional current.  Figure 3(a) shows the variability of TIWs for the 50-year period, which includes the entire range of variability. A large variation occurred between 110°–150°W near the equator, and the maximum value was observed at 4°N.  Figure 3(b) shows the variability of TIWs obtained from the climatological cycle of a TIW, which includes variability that is shorter than a year (hereafter, seasonal variability). The spatial pattern was very similar to that in Figure 3(a), but the amplitudes were smaller and the longitudinal distributions narrower. The same calculations were applied to the temperature and zonal currents. The distributions of variability for the temperature and zonal current (data not shown) were similar to the meridional current in both total and seasonal variability. Two maximum loadings of TIWs commonly appear in all variables and also commonly have a much larger variability of TIWs north of the equator than south of the equator.

To estimate the degree of seasonal phase locking of TIW variation, the ratio of the seasonal variability to the total variability (i.e., pattern in Figure 3(b) divided by that in Figure 3(a)) was computed and is shown in Figure 3(c). If TIWs are considered more likely to be random eddies, their climatological average is expected to be small. However, as seen in Figure 3(c), about 20% of the total variability of a TIW can be explained by its seasonal variability between 125° and 140°W around the equator. This result verifies that the amplitude of a TIW is significantly phase locked to the seasonal cycle.

In order to determine what portion of the unfiltered climatological variation is explained by TIW variation, the ratio of the seasonal variability of TIWs to the seasonal variability of unfiltered data was computed (data not shown). From this calculation, the ratio of an intraseasonal cycle, like TIWs, to the seasonal cycle can be estimated. About 10–20% of the temperature and more than 80% of the meridional current climatological variations are explained by the TIW variability around the equator between 110°–150°W. However, unlike temperature and meridional current, TIW variability occupies a very small portion of climatological variation in the zonal current. This is because zonal current has a stronger seasonal cycle than meridional current [29, 30], and the seasonal variability of an unfiltered zonal current is much larger than the seasonal variability of an unfiltered meridional current. Therefore, zonal current has a very low ratio of TIW variation in climatological variation, while a meridional current that has a weak seasonal cycle shows a very high ratio. Especially near the equator, the variation of TIWs in the meridional current is responsible for over 90% of the seasonal variability. Although the seasonal cycle of temperature is strong and its intraseasonal cycle is also strong, it is different from the zonal current. On the whole, TIWs in the temperature and the meridional current contribute significantly to climatological variation. 

As seen in Figure 3(c), 20% of the total variability of TIWs can be explained by its seasonal variability. In order to clarify the seasonal locking of TIW variability, the mechanisms underlying the generation of TIWs were investigated. It is known that TIWs are generated through barotropic instability caused by the shear of zonal currents, and also baroclinic instability associated with the temperature gradient [4–6]. Two instability mechanisms were estimated from the eddy kinetic energy (EKE) equation [31, 32]. The EKE equation can be derived directly from the momentum equations. The EKE equation is given by

(1) (EKE)t=−v−·∇(EKE)−v′·∇(EKE)−−  v′·∇P′−+Bt+Bc−ε,

where $Bt=-\rho_{0}(\overset{-}{u^{\prime }u^{\prime }}{\overset{-}{u}}_{x}+\overset{-}{u^{\prime }v^{\prime }}\left({\overset{-}{u}}_{y}+{\overset{-}{v}}_{x}\right)+\overset{-}{v^{\prime }v^{\prime }}{\overset{-}{v}}_{y})$, $Bc=-\overset{-}{\rho^{\prime }{gw}^{\prime }}$. In (1), EKE indicates $\rho_{0}\overset{-}{\left(u^{\prime 2}+v^{\prime 2}+w^{\prime 2}\right)}/2$, where the overbars denote the monthly mean, primes denote the TIW components that have been applied to a 50-day high-pass-filter, and *ρ*_0_ is the constant value for the density of water,  1000 kgm^−3^. In (1), *Bt* represents the kinetic energy conversion between mean and eddy flows. If *Bt* is positive, then energy is transferred from mean kinetic energy to eddy kinetic energy through barotropic instability. *Bc* represents the energy conversion between kinetic energy and the available potential energy of eddy flow. A positive *Bc* indicates energy conversion from eddy potential energy to eddy kinetic energy through baroclinic instability. Negative *Bt* and *Bc* values indicate a reduction in eddy kinetic energy. The last term in (1) represents the dissipation of EKE. The first three terms in (1) indicate advective terms of EKE and pressure fluctuations by mean and eddy flow. These advective terms only contribute to redistributing the EKE, so we focus on the production terms *Bt* and *Bc*.

**Figure 4:**
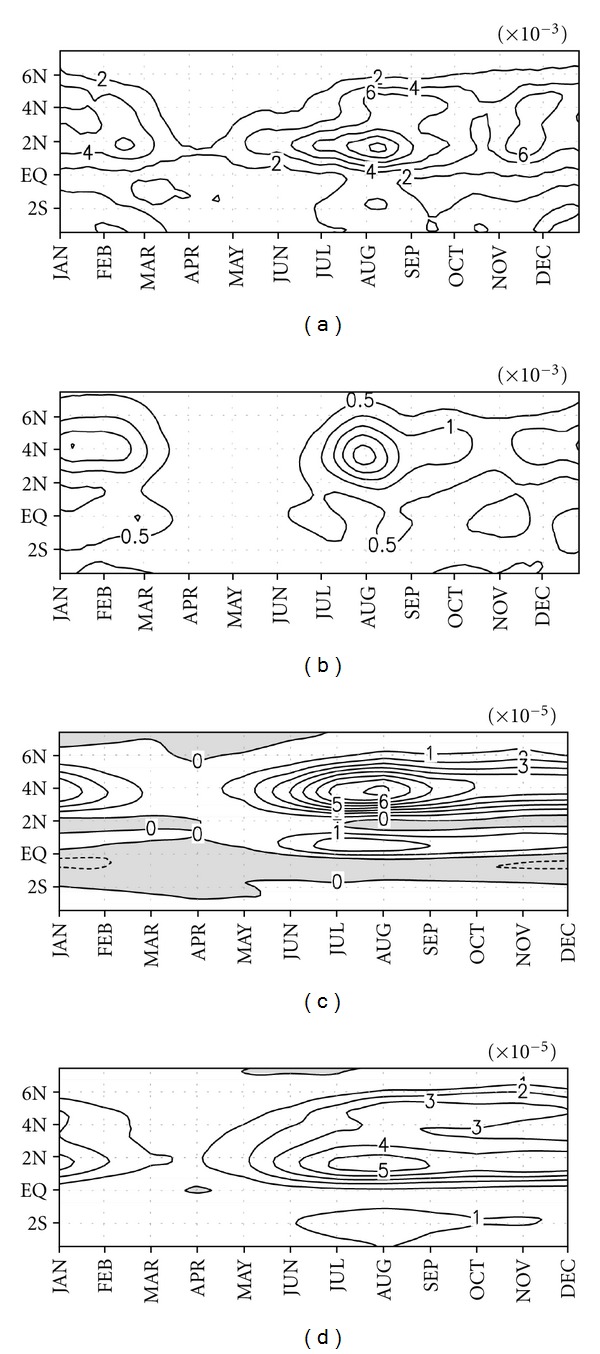
The 50-year climatological mean of the 30-day moving variance of filtered temperature (a) and meridional current (b), and barotropic (c) and baroclinic (d) conversion rates averaged between 160–100°W, and over the upper 50 m. Units are W/m^3^ for (c) and (d). Negative values are shaded.

Figures 4(a) and 4(b) show the 30-day moving variance patterns of temperature and meridional current associated with a TIW, respectively, which were averaged from the surface to a depth of 50 m and over 160°–100°W, where TIW activity is usually strong. The maximum variance of temperature and meridional current appeared at 1°N and 4°N in August, respectively. 

The climatological *Bt* is shown in Figure 4(c), which was averaged over the same domain as in Figure 4(a). *Bt* had a large positive value north of the equator, especially near 4°N, while south of the equator it was much smaller, and became negative at 0°–2°S. North of the equator, the positive *Bt* appeared in June and continued until the end of the year and was maximized during July to August. Under positive *Bt*, TIWs grow by taking kinetic energy from mean flow. Evidently, the variance of the meridional current (Figure 4(b)) becomes strong after June, when *Bt* starts to increase, and, thus, *Bt* is responsible for the growth of a TIW as the dynamical momentum eddy. Positive *Bt* might be attributed to the large meridional shear of NECC and SEC near 4°N and the shear of SEC and EUC near a depth of 80 m between 1° and 2°N. However, our calculation was limited to the upper 50 m, and, thus, the effect of the shear of EUC and SEC, the maximum of which is located around a depth of 80 m, may not significantly contribute to *Bt*. Therefore, between 1° and 2°N, *Bt* may be smaller than it is near 4°N. 

Figure 4(d) shows the climatological *Bc* averaged over the same domain as in Figure 4(c). *Bc* has a positive value almost everywhere, unlike *Bt*. The seasonality of *Bc*, like *Bt*, was dominant. A high value of *Bc* appeared during June–December around 2°N, while it was weakest during March–May. The maximum value of *Bc* appeared during July to August, but remained smaller than *Bt*. *Bc* was large between 1°–2°N and remained relatively small near 4°N where a high *Bt* was recorded. In general, the positive eddy energy production terms are found year-round north of the equator, although it is stronger in the second half of the year. TIWs at 4°N are more associated with barotropic conversion, while TIWs at 0°–2°N are associated with both barotropic and baroclinic conversions that practically excite TIWs as thermal eddies (Figure 4(a)). South of the equator, the EKE of TIWs generated by baroclinic conversion is partly dampened by the negative barotropic conversion effect. Therefore, the EKE of TIWs and related TIW activity were much lower south of the equator. 

**Figure 5:**
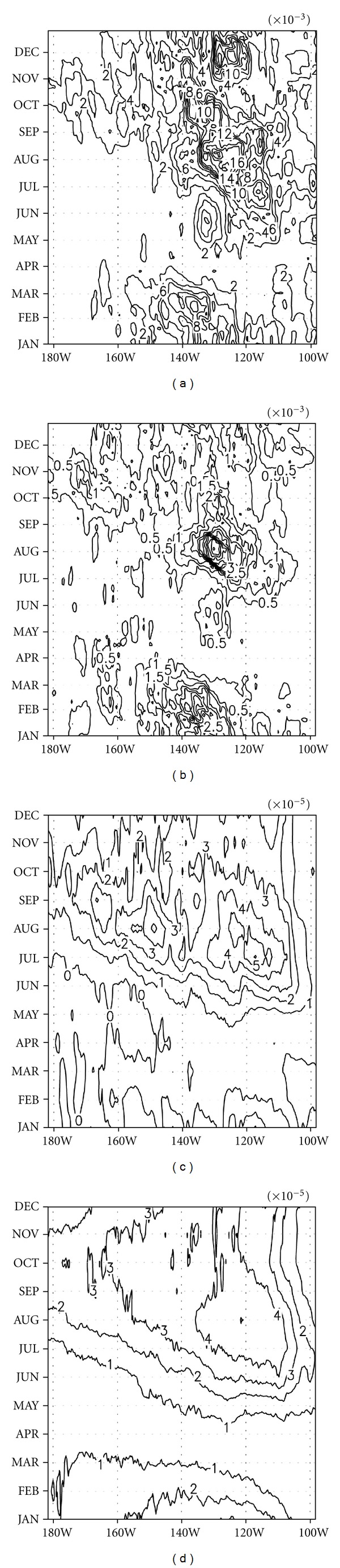
Longitude-time plot of the climatological cycle of the 30-day moving variance of filtered temperature (a) and meridional current (b), and barotropic (c) and baroclinic (d) conversion rates averaged between 0 and 5°N over the upper 50 m. Units are W/m^3^ for (c) and (d).

To depict the longitudinal propagation features of a TIW, *Bt* and *Bc*, a longitude-time plot was drawn of climatological *Bt* and *Bc*, as well as the moving variance of temperature and meridional current, which was used in Figure 4. All values in Figure 5 were averaged between 0°–5°N and a depth of 0–50 m. As shown in Figure 5, TIW activity associated with both temperature and meridional current grew first at the eastern edge of the eastern Pacific around May and spread to the west slowly over time [10]. The maximum activity occurred around 130°W and in August. Consistent features were found in *Bt* and *Bc* as seen in Figures 5(c) and 5(d), respectively, and their contributions were almost equal over this region. 

**Figure 6:**
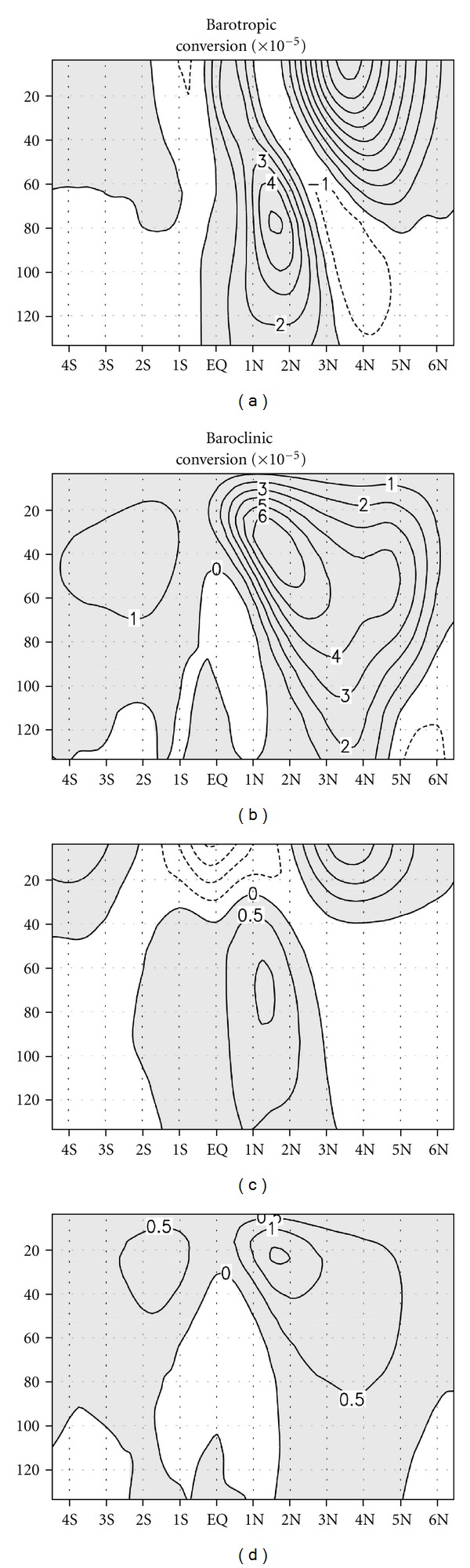
Vertical distributions of zonal mean barotropic ((a), (c)) and baroclinic ((b), (d)) conversions averaged from June to September (a) and (b) and from March to April (c) and (d). Units are W/m^3^. Positive values are shaded.

To illustrate the detailed seasonal features of *Bt* and *Bc*, the zonal averages during the June to September (JJAS) and March to April (MA) seasons were computed, that is, the maximum and minimum TIW activity, respectively. In JJAS (Figure 6(a)), a strong positive conversion was observed near a depth of 80 m between the equator and 3°N, and near the surface between 2° and 6°N. On the other hand, in MA (Figure 6(c)), the maximum but small magnitude of *Bt* was observed only at a depth of 40 m near the surface between 2° and 6°N. Just north of the equator, negative conversion occurred during MA, while a positive conversion occurred during JJAS. Spatial distributions of *Bc* (Figures 6(b) and 6(d)) were similar between the two seasons, but their magnitudes were different. A strong positive conversion appeared just north of the equator, and a weak positive conversion appeared just south of the equator. 

**Figure 7:**
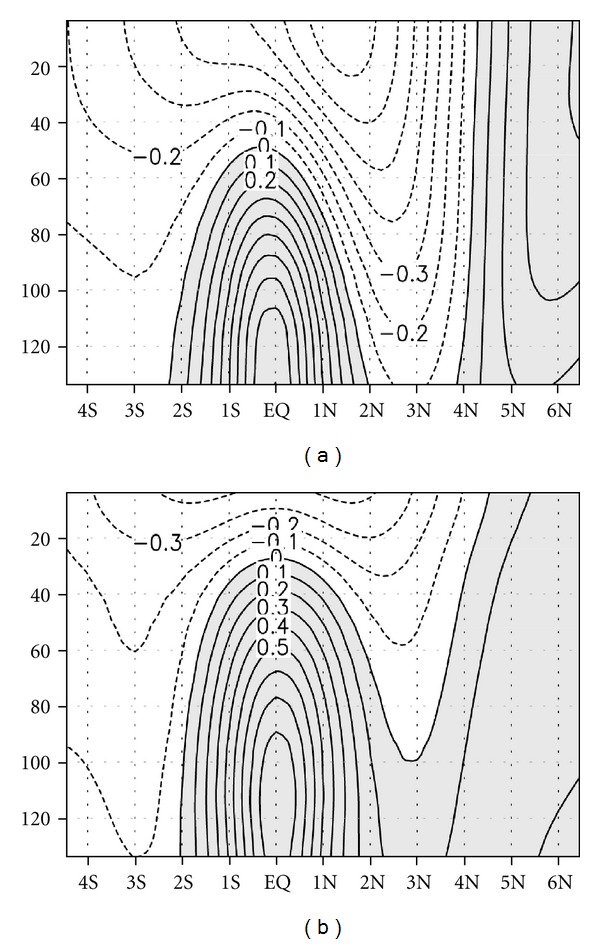
Vertical section of zonal-mean zonal currents averaged (a) from June to September and (b) from March to April, showing the westward SEC (negative contour) and eastward EUC and NECC (positive contour). Units are m/s. Positive values are shaded.

The seasonal variations of *Bt* and *Bc* are related to the seasonality of mean fields.  Figure 7 shows the zonal-averaged zonal currents for JJAS and MA. Between the two seasons, EUC and NECC did not change much, although NECC was somewhat weaker in MA. However, there was a significant change in SEC. The westward SEC had its maximum value during JJAS at 1°-2°N, and its influence reached below 100 m, which produced a large shear between EUC and SEC, inducing a high *Bt*. On the other hand, during MA, SEC became weaker and moved southward such that its influence could not reach the deep layer, and the shear between zonal currents became weaker. Thus, both the strength and position of SEC affect the seasonality of *Bt* thereby influencing the seasonal variation of TIWs. Both zonal and meridional gradients of mean temperature associated with *Bc* were larger in JJAS than in MA, which affects the seasonality of *Bc* and results in the seasonal variation of TIWs.

## 4. Feedback of TIWs to the Seasonal Cycle

The heat budget in the eastern Pacific was analyzed to evaluate the influence of TIWs on the mean temperature change in the tropics. TIWs are stronger from late summer to winter when the cold tongue SST is colder. The contribution of TIWs to ocean temperature can be estimated from anomalous temperature advection by anomalous currents. It is known that temperature advection by TIWs changes the SST budget in the mixed layer [14, 15, 33, 34]. In previous studies, both models [15, 35] and observations [14], showed that horizontal temperature advection by TIWs is as significant, if not more than, mean temperature advection. They also showed that vertical advection by TIWs is small within the mixed layer, whereas it becomes more significant below the mixed layer and induces net cooling. 

**Figure 8:**
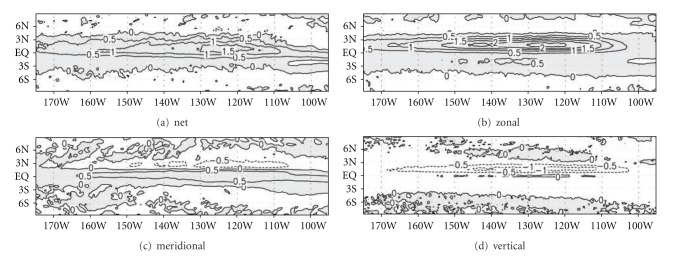
Distributions of temperature advection by TIWs obtained from (a) net advection, (b) zonal advection, (c) meridional advection, and (d) vertical advection. Each advection is averaged from August to December over the upper 50 m. Units are °C/month. Positive values are shaded.

Three-dimensional temperature advection by TIWs was computed in this study. Zonal and meridional temperature advections by TIWs were computed as *u*′*T*_*x*_′ and *v*′*T*_*y*_′, respectively, where primes indicate the TIW components that have been applied to a 50-day high-pass-filter. These zonal and meridional temperature advections were averaged over the depth of 50 m, which roughly represents the depth of the mixed layer. Vertical temperature advection by TIWs was computed as *w*′*T*_*z*_′, which represents heat flux entering the mixed layer by TIW components. *T*_*z*_′ was calculated from the temperature difference between the mixed layer and the subsurface layer located just below the mixed layer. Then, horizontal and vertical advections were added, and climatology was derived from them.  Figure 8(a) shows the spatial distribution of temperature advection by TIWs averaged between August and December, during which TIW activity was strong. The warm advection by TIWs mainly appeared between 4°S and 4°N, and its maximum center was located at 1°N. Outside of 4°S-4°N, the advection by TIWs came into the weak cooling effect. Most of the warm advection was attributed to zonal advection. The meridional advection was smaller than zonal advection, but it was enough to compensate for vertical advection. Thus, in the calculation of the temperature advection by TIWs on the equatorial SST, three-dimensional consideration is necessary. Given the consideration of the seasonal range of equatorial eastern Pacific SST, roughly 3-4 degrees (Figure 1(a)), the warming by TIWs cannot be neglected in the calculation of the seasonal cycle. 

**Figure 9:**
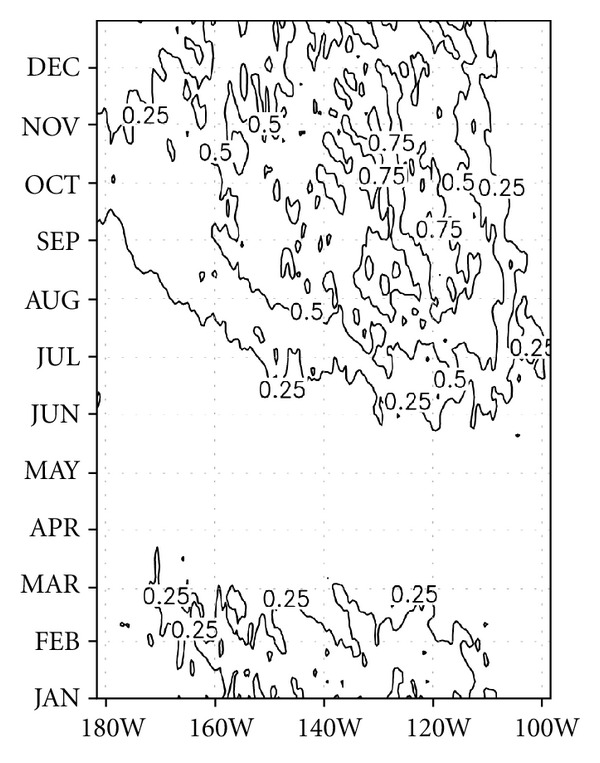
Longitude-time distribution of the climatological cycle of temperature advection by TIWs averaged between 4°S and 4°N from the surface to a depth of 50 m. Units are °C/month. Space smoothing was applied.

The seasonal evolution of the temperature advection by TIWs is shown in Figure 9, in which 4°S-4°N was chosen to average the advection since warm advection is highest in that area. The warm advection began in July near 110°W. The strong advection occurred during September to November, and the maximum value was 1°C/month in September. The annual average of temperature advection by TIWs produced an approximately 0.1–0.5°C/month-warming effect. Therefore, the seasonal cooling of the eastern tropical Pacific during the fall to early winter somehow becomes milder due to the warm advection by TIWs, namely, because of “negative feedback.” 

Since the average temperature variation is about −0.5°C/month in the mixed layer, the temperature advection by TIWs cannot be neglected. This warming is mostly induced by horizontal advection, especially zonal advection, since vertical advection contributes to the cooling effect (see Figures 8(b), 8(c), and 8(d)). In the mixed layer, the warming effect by horizontal advection overcompensates for the cooling effect by vertical advection. However, below the mixed layer, as horizontal advection decreases, the net advection by TIWs decreases due to the compensation between vertical advection and horizontal advection. Thus, the temperature advection induced by TIWs is more effective in the mixed layer than below the mixed layer.

Estimated values of the temperature advection by TIWs from model data were similar to the observation results. Jochum et al., 2007 [14], estimated horizontal temperature advection by TIWs to be 0.8°C/month at 0°N, 140°W and 2.8°C/month at 0°N, 110°W, without vertical advection, whereas our computation was 0.94°C/month warming with −0.02°C/month cooling in vertical advection at 0°N, 140°W, and 1.0°C/month warming with −0.09°C/month cooling in vertical advection at 0°N, 110°W.

## 5. Summary and Concluding Remarks

The climatological cycle of TIWs was analyzed from 50-year ocean model data, which were high-resolution and three-dimensional. The seasonality of TIW activity was represented in its climatological cycle. The results indicate that TIWs are seasonally amplitude-locked such that they are most active during fall and winter and less during spring. Thus, TIW activity is not randomly occurring, but controlled by a change in the mean states. The variability of TIWs is mostly confined between 110° and 150°W near the equator, and its climatological variance explains up to 20% of total TIW variance. In the case of the meridional current, the variability of TIWs in the climatological seasonal cycle comprises more than 80% of the variability of all variations, indicating that the dynamical field activity of a TIW is more strongly locked to a seasonal cycle. 

The seasonality of TIWs has a strong connection with the mean flow, in particular, the meridional shear of the mean flow. From the analysis of barotropic and baroclinic energy conversions, the source of seasonality in TIWs was estimated. The positive correlation between the conversion rate and TIW variability indicates that a strong relationship exists between TIWs and the mean flow and temperature gradient. The heat budget analysis provided insight into how the TIWs influence the seasonal cycle. The temperature advection by TIWs was concentrated in the second half of the year when the activity of TIWs is strong, and it contributes to the change in the mixed layer temperature. TIWs appear to reach down over 500 m, but, on the contrary, the temperature advection by TIWs affects only the upper 50 m. This is because as the depth increases, the positive horizontal advection by TIWs decreases, but the negative vertical advection by TIWs is larger below 50 m, and, thus, they are compensating for each other with depth.

The activity of TIWs is strongly influenced by the cold tongue intensity because of the baroclinic energy conversion associated with temperature gradient. In this regard, the activity of TIWs is associated with the El Nino-Southern oscillation (ENSO) [34, 36]. Therefore, on interannual timescales, the activity of TIWs might be strongest during La Nina when the cold tongue is most pronounced, but weak during El Nino when the SST front is weak [10]. Furthermore, thermal advection by TIWs is greatest during the cold phase of the ENSO cycle, and weakest during the warm phase of ENSO [34, 36]. An and Jin, 2004 [37], suggested that nonlinear dynamical heating could lead to El Nino-La Nina asymmetry, and TIWs were included among them. An, 2008 [34], suggested that thermal heating associated with TIWs can explain the El Nino-La Nina asymmetry based on the results of a simple ENSO model. 

As stated earlier, temperature advection by TIWs was 0.1–0.75°C/month in the climatological cycle, and its effect on the equatorial SST change cannot be ignored. To investigate the effects of TIWs on ENSO asymmetry, analysis to determine the interannual variation of TIWs is necessary. Future work will focus on the interannual variation of TIWs, particularly, the distinct features during an El Nino and La Nina period and their effects on the equatorial SST change from a climatological point of view.
